# Knowledge, Attitudes, and Practices Towards Oral Cancer Among Dental Practitioners in the Northeastern Region of India: An Online Cross-Sectional Survey

**DOI:** 10.7759/cureus.60894

**Published:** 2024-05-23

**Authors:** Happy Riba

**Affiliations:** 1 Oral Medicine and Radiology, Indian Council of Medical Research, Ghaziabad, IND; 2 Oral and Maxillofacial Radiology, ITS Dental College, Ghaziabad, IND

**Keywords:** northeast india, dentists, practice, attitude, knowledge, oral premalignant diseases, oral cancer

## Abstract

Aims: This study aimed to review the overall knowledge of oral cancer based on its clinical presentation as well as associated risk factors, attitudes towards oral cancer examination, and practices among dentists in the northeastern region of India.

Methods and materials: A total of 300 dentists practicing in various parts of the northeastern region participated in this online cross-sectional study. A questionnaire consisting of 26 questions was mailed to the dentists requesting them to fill in the survey form. Based on their responses, knowledge of the risk factors of oral cancer, information on attitudes towards oral cancer examination, and clinical practices were attained.

Statistical analysis: The data was analyzed using IBM SPSS Statistics for Windows, Version 22.0 (Released 2013; IBM Corp., Armonk, New York, United States). Descriptive statistics was used for demographic variables. Unpaired t-test and one-way ANOVA were used to compare the mean knowledge scores with demographic variables. The level of significance was set at P<0.05.

Results: Overall, the knowledge of the risk factors of oral cancer was low among dentists. The majority were not familiar with the proper physical oral examination steps and considered oral medicine specialists to have a vital role.

Conclusions: This study revealed areas in which the knowledge, attitudes, and practices were good to below average and identified areas where improvement is required. Gaps in dentists' knowledge of oral cancer could be identified; dentists displayed substandard knowledge of the clinical presentation as well as risk factors of oral potentially malignant disorders.

## Introduction

Oral cancer is emerging as a major global health emergency. Among other prevalent varieties, oral cancer holds the third position in India as a major cause of mortality and morbidity among the common population [1]. One of the early epidemiological studies assessing the risk of oral premalignant diseases in India reported that 80% of oral cancers were preceded by oral potentially malignant disorders (OPMDs) [2]. The prevalence rate of OPMDs ranges from 1% to 5% worldwide [3].

India and South and Southeast Asian countries have the highest incidence of oral cancer. Squamous cell carcinoma (SCC) constitutes about 90-95% of the oral cancer in India [4]. One study showed that the prevalence of oral squamous cell carcinoma (OSCC) was high in the studied population of Northeast India [5]. Another study showed the peculiarity of cancer incidence pattern in the northeastern region compared to other parts of India in view of the higher proportion of distant metastasis cases at diagnosis, resulting in a comparatively very low survival rate. The desk review stated that the risk of developing tobacco-related cancers (TRCs) is higher in Northeast India [6]. In Northeast India, about 33% of oral cancers are related to the incidence of tobacco [7]. Around 70% of people in Northeast India consume tobacco, as per the National Family Health Survey, which is 26% higher than the national average [8].

To the best of our knowledge, there have been no studies to determine the knowledge, attitudes, and practices among oral clinicians of Northeast India regarding the management of oral pre-cancerous and cancerous lesions. The present study aims to determine the knowledge quotient of regional dental practitioners and their attitudes towards the inclusion of practices related to oral cancer screening in routine examination protocol. The objective of this study was to assess as well as demonstrate the understanding of diagnostic along with risk factors related to oral cancer and further determine their outlook about their proficiency on its prevention and control.

## Materials and methods

This study is a cross-sectional, questionnaire descriptive study carried out in Northeast India from August to October 2020. The study participants were both general dental practitioners and specialists in varied fields of dentistry, functioning in both private and government hospitals and also private setups. Data of 418 dentists, which included name and contact details, who are currently working in northeastern states were acquired through various sources. The questionnaire used in the knowledge, attitudes, and practices study by Lumerman et al. [9] was used in this study (see Appendices). The questionnaire designed in the English language was forwarded via mail to the dentists requesting them to fill in the survey form. All study participants were explained about the purpose of the survey in the mail. Calls were made to enhance the completion rate and encourage them to participate. Out of 418 dentists, 300 reverted to the mail.

The questionnaire consists of 26 questions divided into four sections: (1) clinical practice regarding oral cancer, (2) knowledge of the clinical presentation and risk factors of oral cancer, (3) attitudes towards oral cancer examination, and (4) personal characteristics.

The first section consists of eight questions regarding dental practice. The clinical practice section consisted of three items on the appropriate approach for oral cavity examination, factors that should be probed while taking medical history, and the handiness of educational materials for their patients. The second part consists of 11 questions on the knowledge of the clinical presentation of oral lesions. The attitude section consisted of 11 items meant to estimate the dentists' view regarding their role in oral cancer prevention. There were six items in the personal characteristics section of the questionnaire, consisting of demographic details and the last time they attended a continuing course on oral cancer.

Statistical analysis

The data was analyzed using IBM SPSS Statistics for Windows, Version 22.0 (Released 2013; IBM Corp., Armonk, New York, United States). Descriptive statistics was used for demographic variables. Unpaired t-test and one-way ANOVA were used to compare the mean knowledge scores with demographic variables. The practice of recording different domains of medical history and eliciting risk factors was expressed in proportions. The level of significance was set at P<0.05.

## Results

Personal characteristics

Of the 300 participating dentists, a total of 25 dentists (8.3%) had >15 years of practical experience, and a greater part (194, 64.7%) were general practice dentists. Seventy-two dentists (24%) among the respondents had attended a continuing, or continuous, professional development (CPD) course on oral cancer within the past two years. A significant number of dentists (87%) reported that they have no educational material on oral cancer accessible in their practice. Thirty-two dentists (8%) reported having brochures/pamphlets on oral cancer at hand. Only six dentists (2%) reported that they provide education and awareness on oral cancer. Regarding the detection of the early signs and symptoms of oral cancer, 14% of respondents believed that general practice dentists have the primary role, while 77% considered oral medicine specialists to have a vital role in the detection of oral cancer (Table 1).

**Table 1:** Demographic details CDE: continuing dental education; OC: oral cancer; %: percentage; N: number of frequency

Demographic variables		% (N)
Gender	Male	30 (90)
	Female	70 (210)
Age range	23-29 years	68.3 (205)
	30-39 years	20.3 (61)
	40-49 years	7.3 (22)
	50-59 years	2 (6)
	Above 60 years	2 (6)
Years of practice	<5 years	68.7 (206)
	5-10 years	18.7 (56)
	11-15 years	4.3 (13)
	>15 years	8.3 (25)
Type of practice	General practice	64.7 (194)
	Specialty practice	35.3 (106)
Practice setting	Private clinic	48 (144)
	Hospital setup	52 (156)
Last attended CDE on OC	Within 2 years	24 (72)
	2-5 years	5 (15)
	5-19 years	9 (27)
	Never	62 (186)
When you detect a lesion, you	Refer the patient	82 (246)
	Perform incisional biopsy	13 (39)
	Perform toluidine blue staining	5 (15)
OC patient education material	Always advice verbally	2 (6)
	Brochures/pamphlets	8 (24)
	Videos	3 (9)
	None	87 (261)
Primary role in the detection of OC	ENT specialist	1 (3)
	General dental practitioners	14 (42)
	Oral and maxillofacial surgeons	8 (24)
	Oral medicine specialist	77 (231)

**Figure 1:**
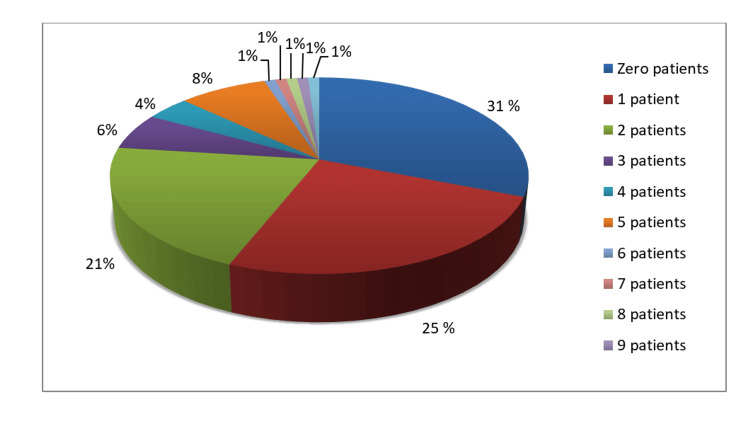
Patients referred for the diagnosis of OC in the last 12 months OC: oral cancer

**Figure 2:**
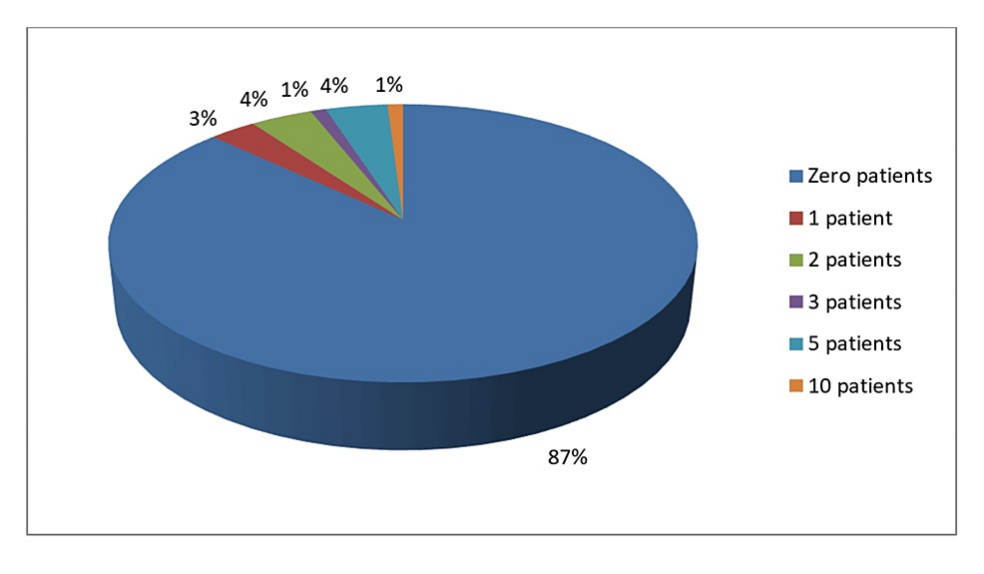
Biopsy performed for OC by study participants in the last 12 months OC: oral cancer

Among the respondents, within the time span of the last 12 months, 69% of dentists had at least referred more than one patient with lesions for the diagnosis of oral cancer. Around 87% of the dentists said they did not perform any biopsy for OPMD in the last 12 months (Figure 1, Figure 2).

Clinical practice regarding oral cancer

**Figure 3:**
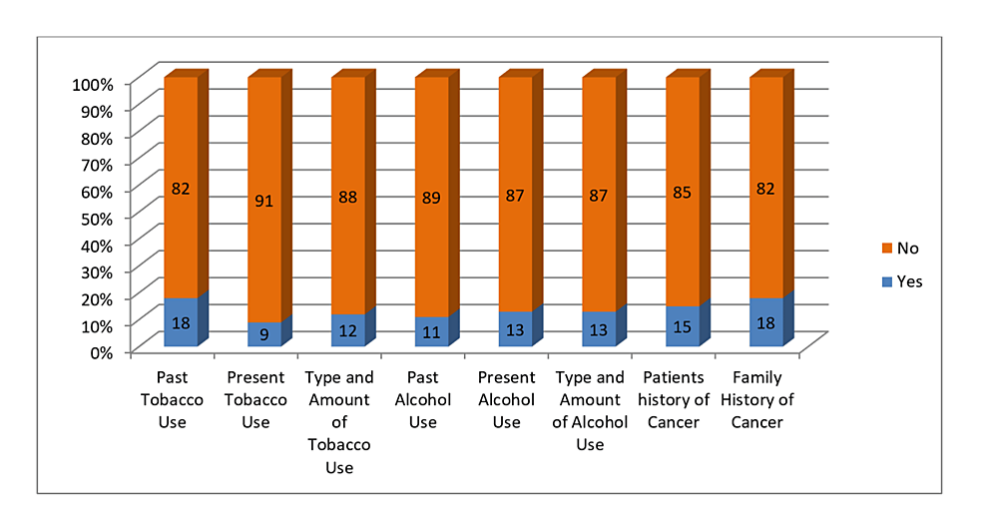
Practice of recording the following in medical history

On evaluation of clinical practice, a larger part of dentists were unversed with the appropriate physical oral examination steps. Moreover, 91% of dentists reported not asking about current tobacco use when taking patient's medical history, 82% didn't ask about previous tobacco use, and 88% reported not inquiring about the type and amount of tobacco used. To a lower extent, dentists reported not asking patients about their current alcohol use (87%), past alcohol use (89%), and type and amount of alcohol used (87%). A small number reported asking about their patients' history of cancer (15%) and family history of cancer (18%) (Figure 3).

Knowledge of the clinical presentation and risk factors of oral cancer

There was no statistically significant difference in the mean knowledge scores among the dentists based on the type of clinical practice (P=00.06). However, those working in a hospital setup had higher mean knowledge scores when compared to those dentists having private clinical practice (P=0.001) (Table 2).

**Table 2:** Mean knowledge scores of dentists with reference to gender, type, and practice setting Level of significance at P<0.05 *statistically significant at P<0.01 using independent sample t-test NS: not significant

	N	Mean	Std. deviation	t	df	P-value
Private clinic	144	2.49	1.3	-3.898	291.08	P=0.001*
Hospital setup	156	3.19	1.7			
General practice	194	2.98	1.6	1.832	298	P=0.06
Specialty practice	106	2.62	1.4			NS

It was observed that those with 11-15 years of clinical practice had higher knowledge scores than the other groups. Similarly, dentists in the age range of 40-49 years had higher mean scores. However, there was no statistically significant difference in the mean knowledge scores with reference to years of practice and age range (Table 3).

**Table 3:** Mean knowledge scores of dentists with reference to their years of practice and age range Level of significance at P<0.05 NS: not significant using one-way ANOVA

	N	Mean	Std. deviation	F	P-value
<5 years	206	2.84	1.646	0.14	P=0.936
5-10 years	56	2.8	1.6		NS
11-15 years	13	3.08	1.038		
>15 years	25	2.96	1.744		
23-29 years	205	2.85	1.645	0.141	P=0.967
30-39 years	61	2.77	1.419		NS
40-49 years	22	3.05	1.864		
50-59 years	6	3	2.449		
More than 60 years	6	3	1.095		

**Figure 4:**
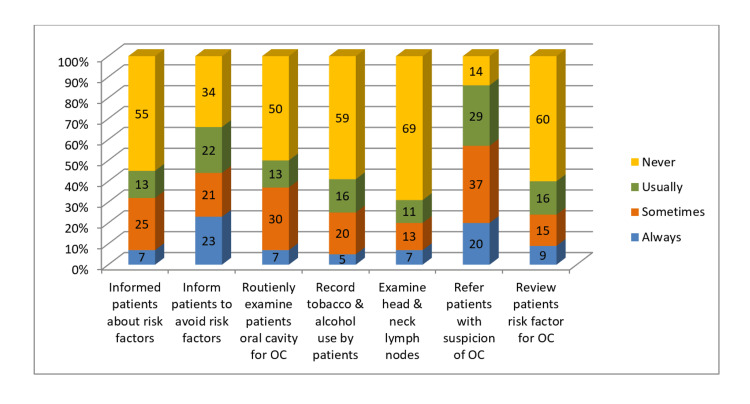
Dentists' practice of eliciting risk factors of OC OC: oral cancer

**Figure 5:**
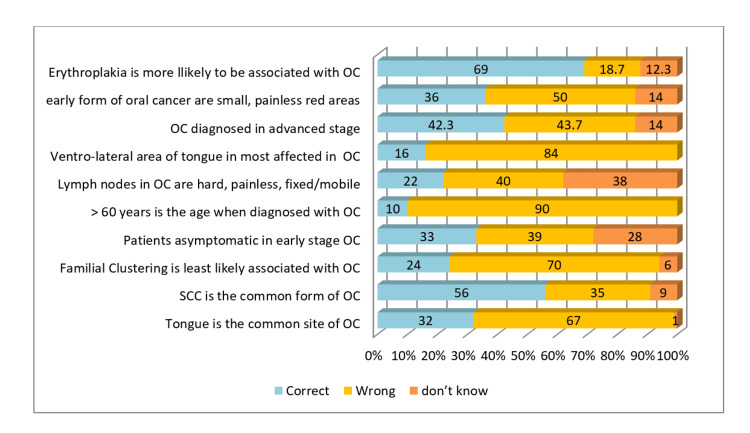
Dentists' knowledge of clinical manifestation of OC (in percentages) OC: oral cancer; SCC: squamous cell carcinoma

The majority of dentists could not correctly identify the tongue as the common site for oral cancer (67%). Over more than half of dentists (56%) correctly replied that SCC is the most common form of oral cancer. Only 33% of dentists answered correctly about early-stage oral cancer being asymptomatic, and 10% correctly answered oral cancer being diagnosed in people aged >60 years in the majority of cases. Around 22% of dentists answered correctly that the lymph nodes are an important site of oral cancer metastasis. Only 42.3% of dentists were familiar that oral cancer in its premalignant form is diagnosed mainly in the early stages. Around 69% of dentists were able to acknowledge erythroplakia as a relatively severe premalignant lesion than leukoplakia (Figure 4, Figure 5).

**Figure 6:**
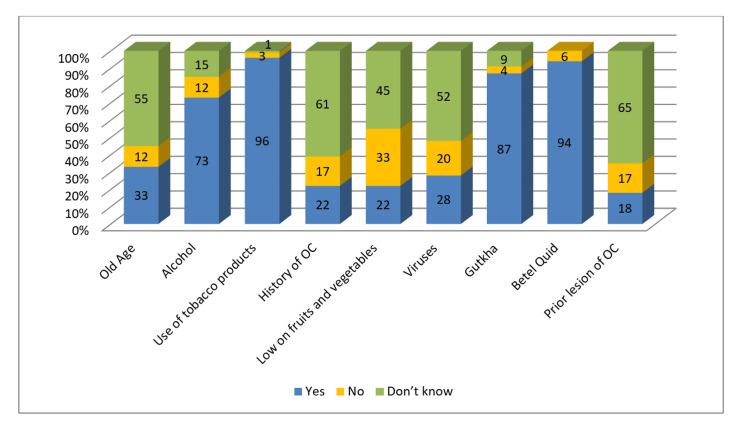
Dentists' knowledge of risk factors of OC OC: oral cancer

A high percentage of dentists correctly identified alcohol (73%), tobacco (96%), gutka (87%), and betel quid (94%) as risk factors for oral cancer. Around 22% of dentists could associate low consumption of fruits and vegetables as a risk factor. This was also seen with older age (33%), viral infection (28%), and prior oral cancer (18%) correctly identifying it as risk factors, while the majority reported that they were unaware of its effect (Figure 6).

**Figure 7:**
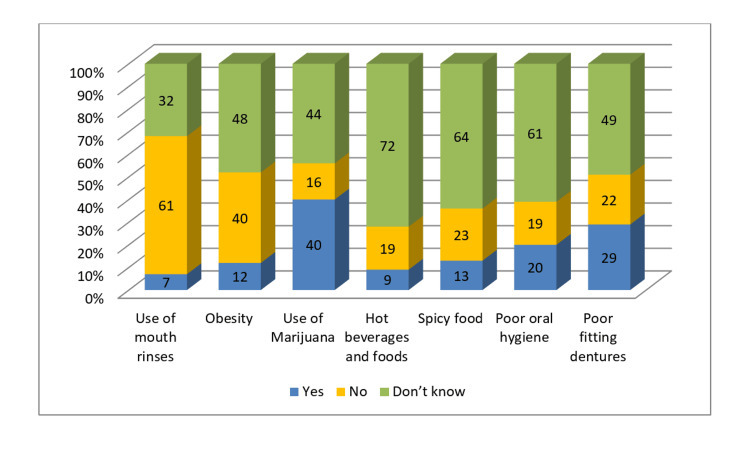
Dentists' knowledge of non-risk factors of OC OC: oral cancer

Mouth rinse use as a non‐risk factor was correctly identified by a larger percentage of dentists (61%). However, a lower proportion could point out spicy food (23%), poor-fitting dentures (22%), and poor oral hygiene (19%) as non‐risk factors (Figure 7).

**Figure 8:**
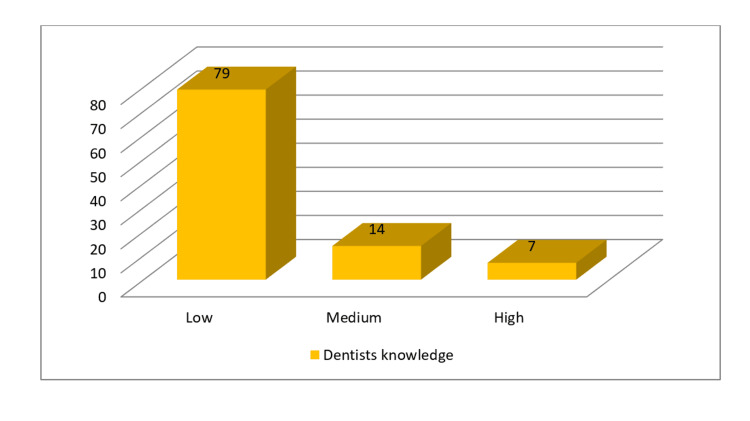
Dentists' overall knowledge of risk factors

Overall, the knowledge of risk factors of oral cancer was low among 79% of dentists. Only 7% had a high knowledge of various risk factors of oral cancer. It was observed that dentists into specialty practice had significantly more knowledge of risk factors than those who were into general practice (P=0.002) (Figure 8, Table 4).

**Table 4:** Association between dentists' characteristics and knowledge of risk factors of OC Level of significance at P<0.05 *statistically significant at P<0.05 and **P<0.01 using the chi-squared test OC: oral cancer

		Knowledge of risk factors			P-value
		Low (n)	Medium (n)	High (n)	
Age range	23-29 years	157	30	18	P=0.598
	30-39 years	49	9	3	NS
	40-49 years	19	3	0	
	50-59 years	6	0	0	
	Above 60 years	6	0	0	
Years of practice	<5 years	158	33	15	P=0.251
	5-10 years	44	6	6	NS
	11-15 years	13	0	0	
	>15 years	22	3	0	
Type of practice	General practice	149	36	9	P=0.002**
	Specialty practice	88	6	12	
Practice setting	Private clinic	114	21	9	P=0.865
	Hospital setup	123	21	12	NS

Attitude towards oral cancer examination

**Figure 9:**
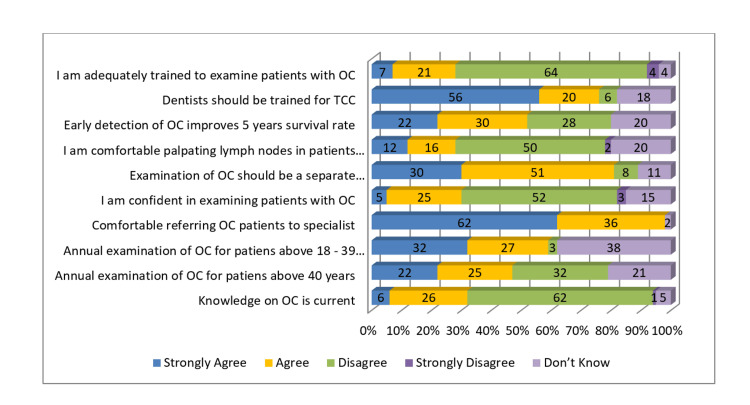
Dentists' attitude towards OC examination (in percentages) OC: oral cancer; TCC: tobacco cessation counseling

About 62% and 64% of dentists strongly disagreed that their knowledge of oral cancer is current and that they are adequately trained to examine patients with oral cancer, respectively. Around 56% strongly agreed that dentists should be trained for tobacco cessation counseling (TCC), and 62% were comfortable referring oral cancer patients to a specialist. Replies regarding dentists' confidence in performing oral cancer examination showed that only 5% of dentists strongly agreed that they were confident in their training. The vast majority of dentists either disagreed or were not sure about the same. More than 50% of dentists agreed that the examination of oral cancer should be a separate reimbursable procedure (Figure 9).

## Discussion

The overall knowledge about oral cancer based on its clinical presentation as well as associated risk factors, attitude towards oral cancer examination, and practices among dentists in the northeastern region of India was determined in the current study. It was found that apart from the dentists, common people displayed poor health-seeking behavior and consideration of the need to undertake cancer screening. This can be attributed to poor overall infrastructure and restricted access to healthcare facilities and inadequacy of trained workforce to conduct the screening program. Results showed a significant shortfall in awareness and cancer prevention programs in the northeastern region.

In the northeastern region of India, the elevated number of tobacco-related OSCC cases is a major concern. This was reflected in our report wherein about 69% of dentists had come across varied number of patients with lesion in the past 12 months and were referred to the specialists for further investigations and treatment. Dentists specifically play a central role in patient awareness and the timely identification of oral cancer. This calls for consciousness about the clinical presentation and risk factors of oral cancer. Overall, the knowledge of risk factors of oral cancer was low among dentists. Although it was observed that dental practitioners into specialty practice had significantly more knowledge on risk factors of oral cancer than those who were into general practice, those working in a hospital setup had higher mean knowledge scores on clinical presentation when compared to those dentists having private clinical practice. Regarding the impact of years of experience, dentists with 11-15 years of clinical experience comparatively showed higher knowledge of the clinical presentation and the risk factors of oral cancer. The majority of dentists were mindful of their obliviousness, hence advocating the necessity of educational interventions for practitioners and dental students.

The present study was conducted to assess the knowledge of risk factor profiles for oral cancer patients among dental practitioners. It was found that a huge proportion of dentists did not inform the patients about the risk factors, and neither did they advise the patients to avoid them. During their clinical practice, a very few dentists agreed that they routinely examined their patient's oral cavity for any malignant or premalignant lesion. This is a mandatory step in the prevention of oral cancer looking at the high prevalence of tobacco habits in the northeastern region despite which proper screening is being neglected.

Dentists are not fully engaged in the prevention and early detection of oral cancer, due to their lack of training. A high proportion of dentists correctly identified tobacco, alcohol, and betel quid use as risk factors for oral cancer. However, it did not reflect in their practice of inquiring patient's medical history, as a higher portion of dentists did not evaluate the patient's tobacco/alcohol use and history of cancer. Apart from tobacco use and alcohol abuse, human pap­illomavirus (HPV) has recently received special attention. HPV-16, in particular, has been linked with the development of a subset of SCC, chiefly at the base of the tongue and the tonsillar area [10]. Knowledge of the effect of HPV was poor among dentists, as a larger part were unaware of HPV as a risk factor for oral cancer. Also, it was found that older age as a potential risk factor for oral cancer development was correctly identified by only 10%. Treated oral cancer patients who continue to smoke have relatively 2-6 times heightened risk of developing a subsequent malignancy of the upper aerodigestive tract than those who quit smoking [11,12]. Very few dentists identified prior lesion of oral cancer as a risk factor. Interestingly, a high proportion of dentists desired to be trained to provide tobacco cessation education. Their knowledge regarding the strong correlation of SCC to tobacco use displayed their eagerness to be engaged in tobacco cessation intervention.

Clearly, the responses from the dentists showed that they had issues in distinguishing between risk and non-risk factors. Dentists who participated in CPD courses on oral cancer within the past two years presumably have knowledge of the clinical presentation and risk factors. Therefore, CPD courses emphasizing on the risk and non-risk factors of oral cancer are imperative. Improving dentists' knowledge of these factors would help to raise mass awareness and consecutively have a positive influence on oral cancer prevention. Additionally, better primary healthcare alongside skilled doctors provides a ground for acknowledging the prospective for the prevention as well as early detection of the disease. 

In dental practice, both extra- and intra-oral examinations are vital steps for a new patient. In this study, a low proportion of dentists correctly identified the involvement of lymph nodes in metastatic cancer. This could be seen reflecting in their attitude, as quite a few dentists admitted being comfortable with examining lymph node. Metastases from OSCC most frequently develop in the ipsilateral cervical lymph nodes. Contralateral or bilateral cervical metastases also can occur, especially in cases of the base of tongue, near the midline, and in advanced oral cancer. Involved nodes usually are enlarged, firm, and non-tender on palpation. In cases of extracapsular spread, the node will feel fixed and immovable. At the time of initial evaluation, as many as 30% of oral cancers have cervical metastases, either palpable or occult [13]. The tongue, in particular, has a rich blood supply and lymphatic drainage, which accounts for the fact that up to 66% of patients with primary tongue lesions have neck disease at the time of diagnosis [14]. A thorough examination of the oral cavity including the tongue comprises an intra-oral examination. A very high proportion of dentists felt that they were untrained for this. Therefore, it is crucial to reinforce continuing education to intensify their basic knowledge and enhance their competency. This could successively boost their confidence and lead to more enthusiastic comprehensive oral examinations for their patients.

SCC as the most common form of oral cancer was correctly recognized by a high percentage of our dentists. Nonetheless, some important constraints in knowledge regarding the clinical presentation of oral cancer were pointed out. A lower percentage of dentists could identify the tongue as the most common site of oral cancer in our study. Bai et al. in their study reported regarding the anatomical distribution of oral cancer in decreasing the frequency of occurrence as follows: tongue, gingiva, buccal mucosa, oral floor, oropharynx, lip, and palate [15]. Neville and Day in their study also stated that the most common site for intra-oral carcinoma is the tongue, most frequently the posterior lateral border and ventral surfaces of the tongue accounting for around 40% of all cases in the oral cavity proper followed by the floor of the mouth [16]. Very few dentists who participated in this study correctly answered the ventrolateral surface as the most affected area of the tongue. Two major factors have been explained as why this region is at high risk: firstly, carcinogens that mix with saliva pool in the bottom of the mouth and are constantly in contact with these sites, and secondly, the presence of thinner, non-keratinized mucosa, which provides less protection against carcinogens [17]. It is paramount for the clinician to be aware of this vulnerable region during intra-oral examination.

Very early pre-clinical invasive cancers, i.e., early-stage cancers without symptoms, present as painless small ulcers, nodular lesions, or growths. Less number of dentists correctly responded that during its early stages, oral cancer is asymptomatic. Accordingly, outnumbered dentists responded that OC is predominantly diagnosed at advanced stages. Fortunately, nearly half of the dentists were aware of erythroplakia and leukoplakia as indications of a premalignant lesion, and 69% of dentists correctly identified erythroplakia as a more serious premalignant lesion. These are characteristic signs of premalignancy that every dentist is obliged to know.

In the northeastern region of India, most patients with cancer present with advanced-stage disease, primarily because of delayed presentation and subsequent diagnosis and treatment which is often inadequate, resulting in high mortality rates. This can be inferred from the fact that the five-year survival rate of head and neck cancer for the rest of India is 74%, while in the northeastern region, it is just 40% [18]. Reduction in the five-year survival rate of patients with oral cancer from 80% for those diagnosed with early-stage cancer to 20% for those diagnosed at advanced stages accentuated the call for early detection and comprehensive treatment [19]. Since the five-year survival is directly related to the stage at diagnosis, prevention, and early detection, efforts have the potential not only for decreasing the incidence but also for improving the survival of the patient. An astute clinician or patient is responsible for the early diagnosis of suspicious lesion and symptom at their very initial stage. However, it is evident that a majority of dentists have inadequate knowledge about the risk factors, diagnosis, and early detection of these cancers and/or are not implementing routine examinations [10,20]. Therefore, in order to improve survival rate, public awareness efforts are also imperative to encourage patients to avoid indulging in high-risk behavior and seek regular oral cancer screening examinations. By implementing screening programs for the early detection of oral cancer, the high mortality rate can be drastically reduced [21,22].

Oral medicine specialists are specially trained for the diagnosis and management of such lesions. Interestingly, majority of the dentists considered the primary role of oral medicine specialists in the detection of malignant as well as premalignant oral diseases. Also, a high proportion of dentists preferred referring patients to a specialist upon coming across any such lesion.

One of the limitations of this study is non-response bias. Indeed, non-respondents to this survey might have a different level of knowledge than respondents. However, there were respondents from all groups based on age, type of practice, and years of experience, and also, dentists from various specialties were included; hence, we believe there is no major difference between the respondents and non-respondents.

## Conclusions

The available infrastructure is distinctly inadequate, despite the heavy burden of cancer in the region; hence, people in the northeastern region of India are compelled to seek treatment outside the state. The Rural Health Statistics of the Ministry of Health Family Welfare 2014-2015 reported that 78.7% of cancer patients are seeking treatment outside the region. The current survey reveals the need for oral medicine specialists in these regions. As the burden of oral cancer is increasing, it is important to detect the disease at an early stage and help in increasing the survival rates and reducing morbidity and mortality from these cancers. Early detection is the utmost critical intervention impacting the survival of oral cancer patients. Also, further training of dental professionals is necessary to combat oral cancer and its associated risk factors and to emphasize the practitioners' propensity in diagnosing OPMD.

## Figures and Tables

**Figure 10:**
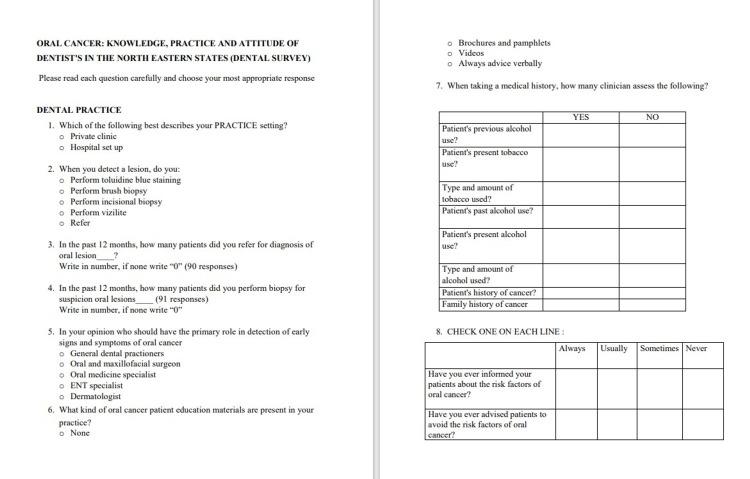
Questionnaire

**Figure 11:**
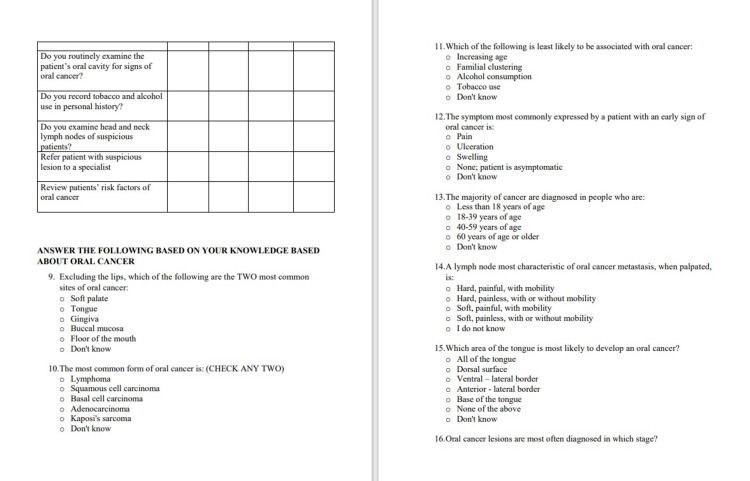
Questionnaire (Contd.)

**Figure 12:**
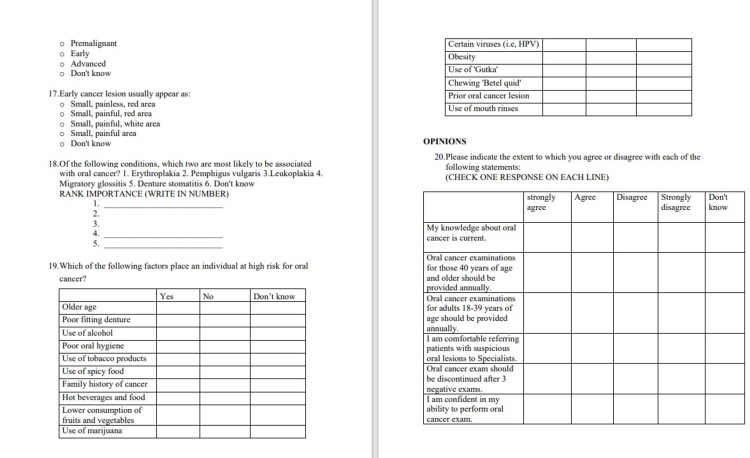
Questionnaire (Contd.)

**Figure 13:**
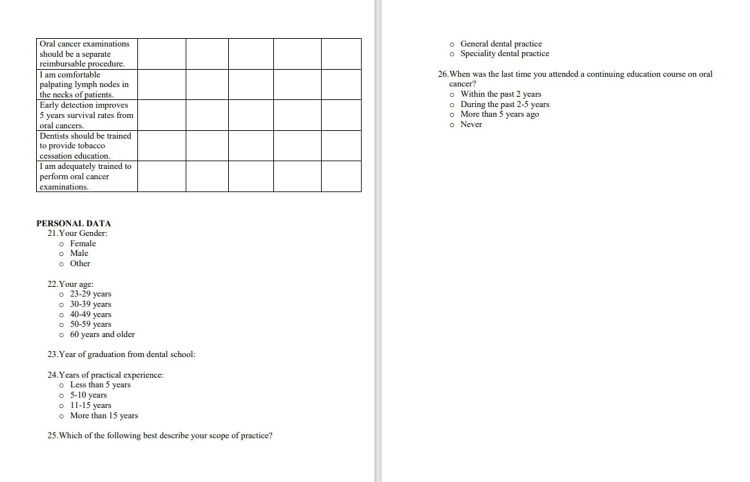
Questionnaire (Contd.)

**Figure 14:**
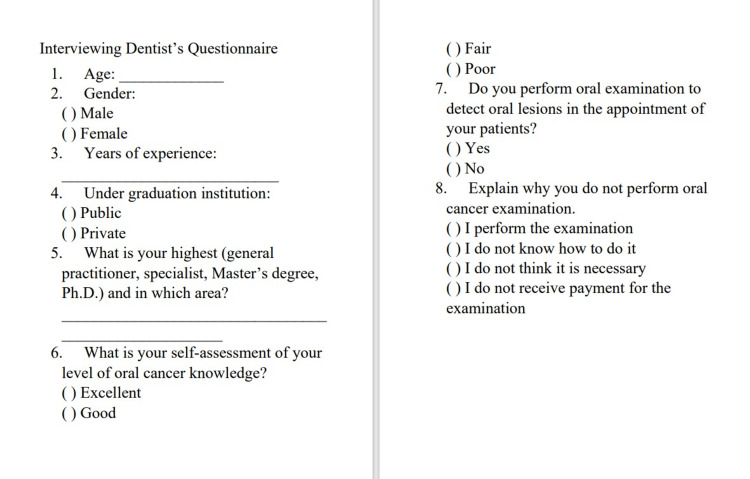
Questionnaire (Contd.)
